# Data-driven identification of core tumor-secreted factors associated with cachexia prevalence

**DOI:** 10.1016/j.gendis.2025.101883

**Published:** 2025-10-17

**Authors:** Sarah Santiloni Cury, Paula Paccielli Freire, Robson Francisco Carvalho

**Affiliations:** a Department of Structural and Functional Biology, Institute of Biosciences, São Paulo State University (UNESP), Botucatu, SP 18618-689, Brazil; b Department of Genetics, Microbiology, and Immunology, Institute of Biosciences, São Paulo State University (UNESP), Botucatu, SP 18618-689, Brazil; c Department of Immunology, Institute of Biomedical Sciences, University of São Paulo, SP 05508-000, Brazil; d Department of Clinical and Toxicological Analyses, School of Pharmaceutical Sciences, University of São Paulo, SP 05508-000, Brazil; e Department of Cell and Developmental Biology, Institute of Biomedical Sciences, University of São Paulo, SP 05508-000, Brazil

Secreted components from the tumor microenvironment actively drive the development and progression of cancer cachexia. These cancer cachexia tumor factors (CCTFs) trigger systemic inflammation, metabolic alterations, skeletal muscle wasting, and adipose tissue lipolysis. Many of these factors are members of the cytokine–cytokine receptor interaction (CCRI) pathway, which is particularly enriched in tumor types highly associated with cachexia. For example, pancreatic ductal adenocarcinoma (PDAC) has the highest prevalence of cachexia and exhibits the highest number of overexpressed secreted genes. Our previous pan-cancer study demonstrated that secretory genes (tumor secretome) belonging to the CCRI pathway are up-regulated in tumors highly associated with cachexia.^1^ PDAC expressed the highest number of secreted genes from the CCRI pathway. Considering the potential of this pathway to have other CCTFs not previously associated with cachexia, it is crucial to determine a core of genes representative of the pathway in the context of cancer cachexia. Moreover, we expect to distinguish the primary source of CCTF by analyzing tumor transcriptomes (bulk and single-cell). Therefore, we aimed to investigate the secretome genes related to the CCRI pathway in 12 cancer types with different prevalences of cachexia, thereby generating what we called the core of cancer cachexia tumor factors (core-CCTF).

Using a pan-cancer approach, we evaluated secreted CCTF genes in 12 cancer types with varying cachexia prevalence (4651 TCGA samples from BRCA: invasive breast carcinoma; COAD: colon adenocarcinoma; ESCA: esophageal carcinoma; HNSC: head and neck squamous cell carcinoma; LAML: acute myeloid leukemia; LIHC: liver hepatocellular carcinoma; LUAD: lung adenocarcinoma; LUSC lung squamous cell carcinoma; PAAD: pancreatic adenocarcinoma; PRAD: prostate adenocarcinoma; READ: rectal adenocarcinoma; STAD: stomach adenocarcinoma) compared with matched normal tissues (2737 GTEx samples). First, we selected 263 genes from the CCRI pathway and predicted as secreted by The Human Protein Atlas. We identified 71 genes up-regulated in PDAC and shared with at least one cancer type. Most of these genes are highly expressed in cachexia-associated cancers and absent/down-regulated in cancer types where cachexia is not prevalent (Fig. 1A). We denominated this gene set as a core-CCTF. Through Pearson correlation-based clustering analysis, we identified a distinct co-expression module comprising CCTF with the highest positive correlations, which also shows increased expression in tumor types with higher cachexia prevalence (Fig. S1), suggesting a coordinated transcriptional program potentially associated with cachexia-related mechanisms. To understand how these molecules interact, we constructed a protein–protein interaction network with 26 core-CCTF genes up-regulated in PAAD, STAD, READ, COAD, ESCA, and HNSC (cancer types highly associated with the syndrome). This indicates a potential use of the described genes as a panel of biomarkers for cachexia risk stratification or potential therapeutic targets. Indeed, C-X-C motif chemokine ligand 8 (*CXCL8*), also known as interleukin-8 (IL-8), has the highest centrality and interacts with other genes related to the chemokine interleukin-8-like superfamily (Fig. 1B). IL-8 is a tumor-derived factor directly associated with muscle wasting in lung and pancreatic cancers, and its neutralization with blocking antibodies prevented *in vitro* muscle atrophy (refer to the supplementary materials for references).

**Figure 1:**
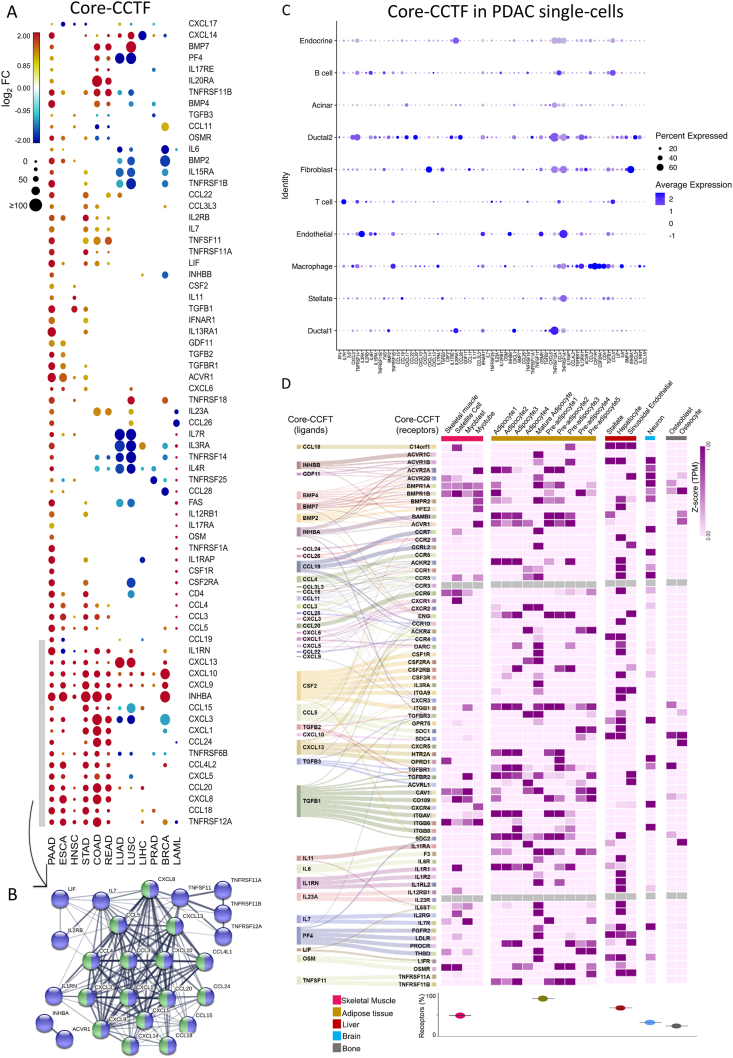
The cytokine–cytokine receptor interaction (CCRI) pathway's gene expression landscape reveals a core of coding proteins predicted as secreted, enriched in cancer, and highly associated with cachexia. **(A)** A heat-scatterplot of 71 secretory genes from the CCRI pathway shared with PAAD for each cancer type (the core of cancer cachexia tumor factors (core-CCTF). The circles' color corresponds to the log_2_fold-change, while the circle size is based on the –log_10_ adjusted *p*-value in the tumor compared with the corresponding healthy tissues. Rows and columns were clustered based on Euclidean distance. Differential expression levels were calculated using the web-based tool Gene Expression Profiling Analysis (http://gepia.cancerpku.cn/). The up-regulated and down-regulated genes with absolute values of |fold change| > 2.0 and *Q*-value <0.01 are shown in red and blue, respectively. The resulting numbers of genes encoding predicted secreted proteins were filtered based on the Human Protein Atlas secretome data (https://www.proteinatlas.org/humanproteome/secretome). BRCA, breast invasive carcinoma; COAD, colon adenocarcinoma; ESCA, oesophageal carcinoma; HNSC, head and neck squamous cell carcinoma; LAML, acute myeloid leukaemia; LIHC, liver hepatocellular carcinoma; LUAD, lung adenocarcinoma; LUSC, lung squamous cell carcinoma; PAAD, pancreatic adenocarcinoma; PRAD, prostate adenocarcinoma; READ, rectum adenocarcinoma; STAD, stomach adenocarcinoma. **(B)** From the up-regulated genes identified in (A), protein–protein interactions (PPI) were identified in STRING of secreted proteins coded by 26 up-regulated genes shared by six cancer types highly associated with cachexia (PAAD, ESCA, STAD, HNSC, COAD, and READ). Edge thickness indicates confidence. Interacting proteins from the CCRI pathway are represented in blue and correspond to a gene set of 26 out of the 263 genes constituting this pathway. We also observed a central subcluster in this PPI pathway enriched by 16 proteins of the chemokine interleukin-8-like superfamily pathway, represented in green. Only proteins significant in at least four tissue sites are represented in the PPI pathway. PPI was created using STRING tool v.11 (https://string-db.org/) and Cytoscape (v3.7.2). The arrow indicates the up-regulated genes used to generate the PPI. **(C)** Dotplot with expression values of core-CCTF genes from (A) in 57,530 pancreatic cells using single-cell RNA sequencing data (GSA: CRA001160) from PDAC and normal-like pancreatic tissues (*n* = 27 and *n* = 14, respectively). **(D)** Inference of the crosstalk between tumor cells and tissue cells affected by cachexia using core-CCTF gene expression data. Left: Alluvial diagram connecting the ligands from the core of factors (CCTF) into its matched receptors (data from Ramilowski et al). Right: Heat map of the expression levels (z-score normalized Transcripts Per Million - TPM) of predicted receptors in human primary cell lineages, previously described by Ramilowski et al^3^ using the FANTOM5 project. Bottom: The scatter plot showing the percentage of core-CCTF receptor genes expressed in each tissue. Muscle: 31 out of 71 receptors (43.7%); Adipose tissue: 60 out of 71 receptors (85.5%); Liver: 44 out of 71 receptors (62%); Brain: 22 out of 71 receptors (31%); Bone: 13 out of 71 receptors (18.3%). Adipocyte1, breast adipocyte; Adipocyte2, omental adipocyte; Adipocyte3, perirenal adipocyte; Preadipocyte1, breast preadipocyte; Preadipocyte2, omental preadipocyte; Preadipocyte3, perirenal preadipocyte; Preadipocyte4, subcutaneous preadipocyte; Preadipocyte5, visceral preadipocyte.

Since PDAC was the tumor type with significant up-regulation of the 71 core-CCTF genes, we further focused on this tumor type and this gene list for the subsequent analyses. We found that gene amplification is a genomic alteration most found on core-CCTF genes, present in 217 out of 988 PDAC patients from cBioPortal (Fig. S2A). These patients with alteration in core-CCTF genes have higher scores of tumor hypoxia (Fig. S2B) and lower disease-free survival rates (Fig. S2C; *n* = 69).

Further, we analyzed the public single-cell RNA-sequencing data from pancreatic cancer samples (CRA001160)^2^ to characterize the expression landscape of core-CCTF genes. We found that macrophages, B cells, and T cells were most abundant in cancer tissues and reduced in the normal pancreas (Fig. S3A and S3B). T cells and macrophages were also increased in TCGA PDAC samples compared with GTEx normal pancreas, as demonstrated by the CIBERSORTx deconvolution tool (Fig. S3C). Sixty-nine core-CCTF genes were available in the single-cell analysis. Overall, malignant ductal cells (Ductal2) express 57.9% of core-CCTF genes, followed by macrophages (39.1%), fibroblasts (39.1%), and T cells (27.5%) (Fig. 1C; Fig. S3D). Normal ductal cells (Ductal1) express fewer core-CCTF genes (29%) when compared with the neoplastic cells (Fig. 1C). We verified that the majority of genes were expressed by higher percentages in malignant cells (25%–87%; Fig. S4). *CXCL5* (78.93%), C–C motif chemokine ligand 15 (*CCL15*) (85.54%), and platelet factor 4 (*PF4*) (87.03%) were particularly expressed in malignant ductal cells (Fig. S3D; Fig. S4). Liu et al have shown that macrophages potentiate pancreatic cancer-induced muscle wasting by promoting tumor necrosis factor (TNF)-like weak inducer of apoptosis (TWEAK) secretion from tumor cells, and that depletion of macrophages can reverse tumor-driven muscle degradation. Macrophages activate malignant cells through the CCL5/TNF receptor-associated factor 6 (TRAF6)/nuclear factor-kappa B (NF-κB) pathway, leading to non-autonomous secretion of TWEAK (refer to the supplementary materials for reference). This evidence supports a model in which malignant epithelial cells and macrophages act cooperatively to sustain a pro-cachectic signaling network, consistent with our finding that both cell types are central sources of core-CCTFs in PDAC. We validated the increased expression of TNF receptor superfamily member 1A (*TNFRSF1A*), *TNFRSF6B*, *TNFRSF12A*, *TNFRSF14*, *TNFRSF25*, *FAS*, Bone morphogenetic protein 4 (*BMP4*), activin A receptor type 1 (*ACVR1*), interferon alpha and beta receptor subunit 1 (*IFNAR1*), interleukin 15 receptor subunit alpha (*IL1*5RA), interleukin 1 receptor accessory protein (*IL1RAP*), interleukin 4 receptor (*IL4R*), oncostatin M receptor (*OSMR*), leukemia inhibitory factor (*LIF*), transforming growth factor beta 1 (*TGFB1)*, *TGFB2*, *CXCL1*, *CXCL8*, and *CXCL5* in pancreatic cancer cell lines from the Cancer Cell Line Encyclopedia (Fig. S5). The PDAC cell line PANC0213 presented the most enriched profile for core-CCTF genes (Fig. S5), suggesting that this cell line is the most similar to PDAC cancer cells in terms of the transcriptional profile of CCTF genes.

To infer the crosstalk between tumor cells and tissues affected by cachexia, we used the ligand-receptor consensus list by Ramilowski et al^3^ to verify the expression of the CCTF receptors in human primary cells from the FANTOM5 project. We observed that adipose cells (mature adipocytes), followed by liver cells (hepatocytes), express the highest number of receptor genes (85.5% and 62%, respectively) for the core-CCTFs expressed in the tumors highly associated with cachexia (Fig. 1D). Neurons and bone cells express the lowest number of core-CCTF receptor genes (31% and 18.3%, respectively). Skeletal muscle cells express 43% of core-CCTF receptor genes. This may indicate that adipose tissue is more responsive to cachexia stimulus than skeletal muscle. A previous study has shown a more pronounced transcriptomic response in the subcutaneous adipose tissue compared with the rectus abdominis muscle in PDAC cachectic patients.^4^ The receptors C-X-C motif chemokine receptor 1 (CXCR1), CXCR2, CXCR3, C–C motif chemokine receptor 5 (CCR5), and CCR1 presented more than three ligands from the core-CCTF list, as demonstrated in the KEGG database (Fig. S6A). In a pancreatic cancer cachexia model (GSE51931),^5^ *Ccr1* and *Ccr5* were up-regulated (Log_2_fold-change > 1.15 and *p*-value < 0.05) in skeletal muscle, white adipose, and liver tissues (Fig. S6B). *Cxcr2* was only significantly overexpressed in the liver of cachectic animals (Fig. S6B).

Overall, we identified a core of secreted factors associated with the CCRI pathway, specifically enriched in tumor types with a high prevalence of cachexia. By analyzing bulk and single-cell data from PDAC samples, the cancer type with the highest risk of inducing cachexia, we found increased immune cellularity (number and diversity) compared with normal samples. We also found that malignant ductal cells were responsible for the expression of most genes belonging to the core, followed by stromal and immune cells. Moreover, we found that the core-CCTF genes coded proteins able to interact with muscle cells, hepatocytes, and adipocytes via specific receptors expressed in these cells, especially in mature adipocytes. These results show novel potential pro-cachectic mediators that can trigger wasting in the targeted tissues most affected in cancer cachexia.

## CRediT authorship contribution statement

**Sarah Santiloni Cury:** Writing – review & editing, Writing – original draft, Validation, Methodology, Investigation, Formal analysis, Data curation. **Paula Paccielli Freire:** Writing – review & editing, Visualization, Methodology, Investigation, Formal analysis, Data curation. **Robson Francisco Carvalho:** Writing – review & editing, Writing – original draft, Supervision, Resources, Project administration, Methodology, Investigation, Funding acquisition, Conceptualization.

## Data availability

The results shown here are whole based upon data generated by the TCGA Research Network (http://cancergenome.nih.gov/) and by the Genotype-Tissue Expression project (GTEx) (https://gtexportal.org/). The results are also partly based on publicly available microarray data from the Gene Expression Omnibus (GEO) and single-cell RNA-Seq dataset from the Genome Sequence Archive (GSA): GSE51931 and CRA001160, respectively.

## Declaration of generative AI and AI-assisted technologies in the writing process

During the preparation of this work the author(s) used Grammarly and ChatGPT 4 in order to improve language and readability. After using this tool/service, the author(s) reviewed and edited the content as needed and take(s) full responsibility for the content of the publication.

## Funding

We acknowledge the financial support for this research provided by the São Paulo Research Foundation (Brazil) (No. 2020/03854-4 to R.F.C.) and by the National Council for Scientific and Technological Development (Brazil) (No. 311603/2022-0 to R.F.C.; 141601/2019-1 to S.S.C.). This study was also partially financed by the Coordination for the Improvement of Higher Education Personnel (CAPES) - Finance Code 001, Brazil.

## Conflict of interests

The authors have read the journal's authorship agreement and declare no conflict of interests.

## Figures and Tables

**Figure S1:**
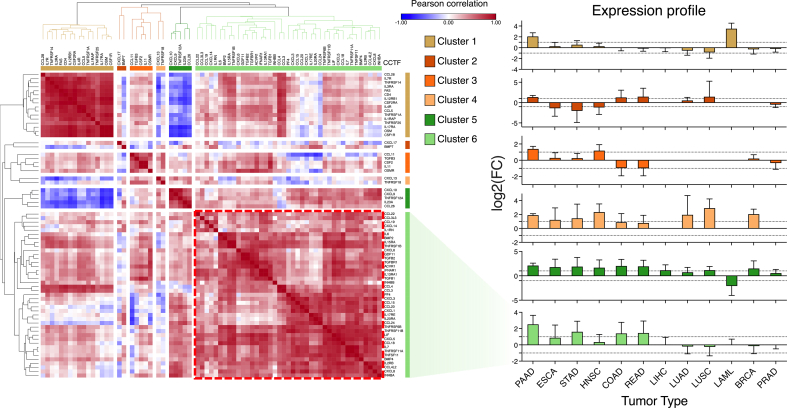
**Pearson correlation-based clustering analysis of core-CCTFs and expression profiles across tumor types.** The heatmap on the left shows co-expression modules identified among the 71 core-CCTFs, with the highlighted clusters representing groups of genes with the highest correlations. On the right, the bar plots represent the average log2 expression values of each cluster across the 12 analyzed tumor types, revealing distinct transcriptional patterns associated with cachexia prevalence.

**Figure S2:**
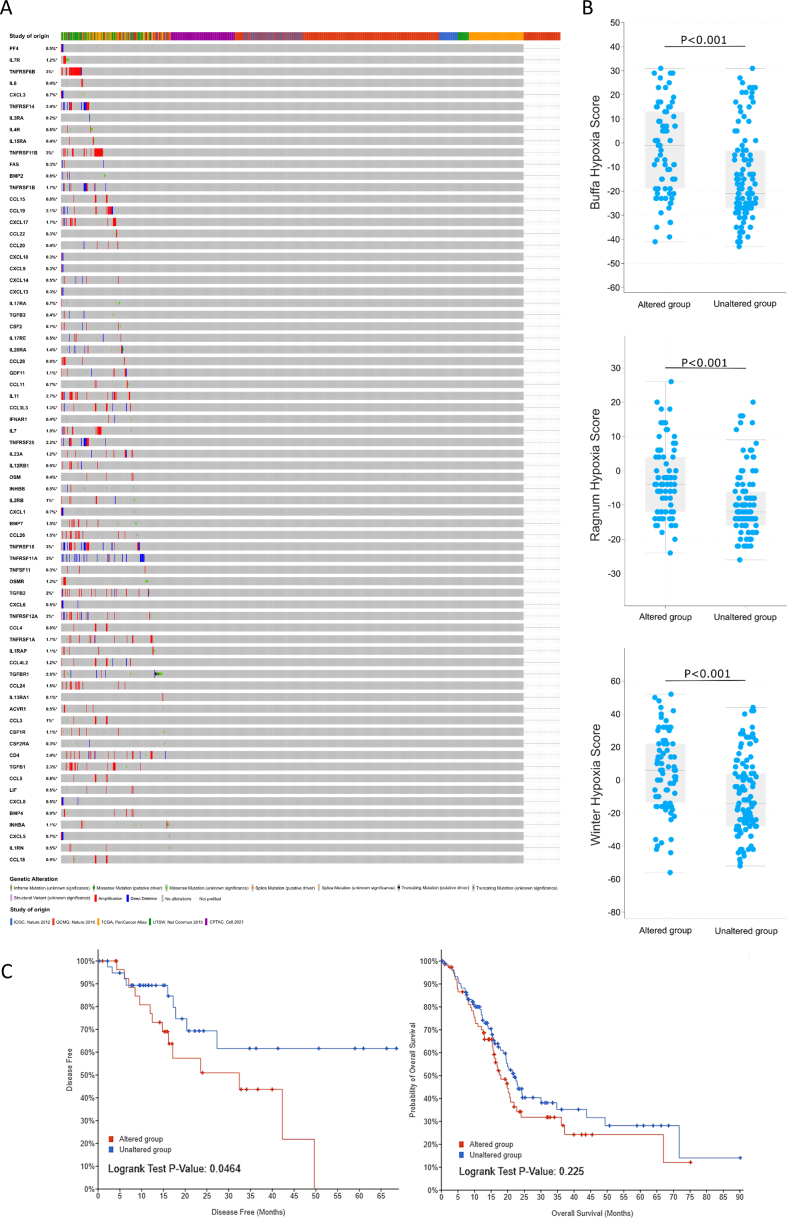
**Genomic alterations on core-CCTF genes are associated with high hypoxia scores and lower disease-free survival. A)** Oncoprint demonstrating genomic alterations of 71 core-CCTF genes in 988 pancreatic adenocarcinoma patients. **B)** Box-plot representing hypoxia scores calculated by the cBioPortal tool using three metrics (Buffa, Ragnum, and Winter). The statistical significance was analyzed using the Wilcoxon test. P-values are corrected using the Benjamini-Hochberg FDR procedure. **C)** Kaplan-Meier disease-free survival curve for 69 PDAC patients (left). The resulting p-values for the log-rank test are shown. Kaplan-Meier overall survival curve for 189 PDAC patients (right). The resulting p-values for the log-rank test are shown. Altered group: PDAC patients with gene alterations in core-CCTF genes. Unaltered group: PDAC patients with wildtype core-CCTF genes. Figure generated using cBioPortal web tool (https://www.cbioportal.org/) using patients’ available information from five different databases: International Cancer Genome Consortium (ICGC), Queensland Centre for Medical Genomics (QCMG), The Cancer Genome Atlas (TCGA), University of Texas Southwestern (UTSW), and Clinical Proteomic Tumor Analysis Consortium (CPTAC).

**Figure S3:**
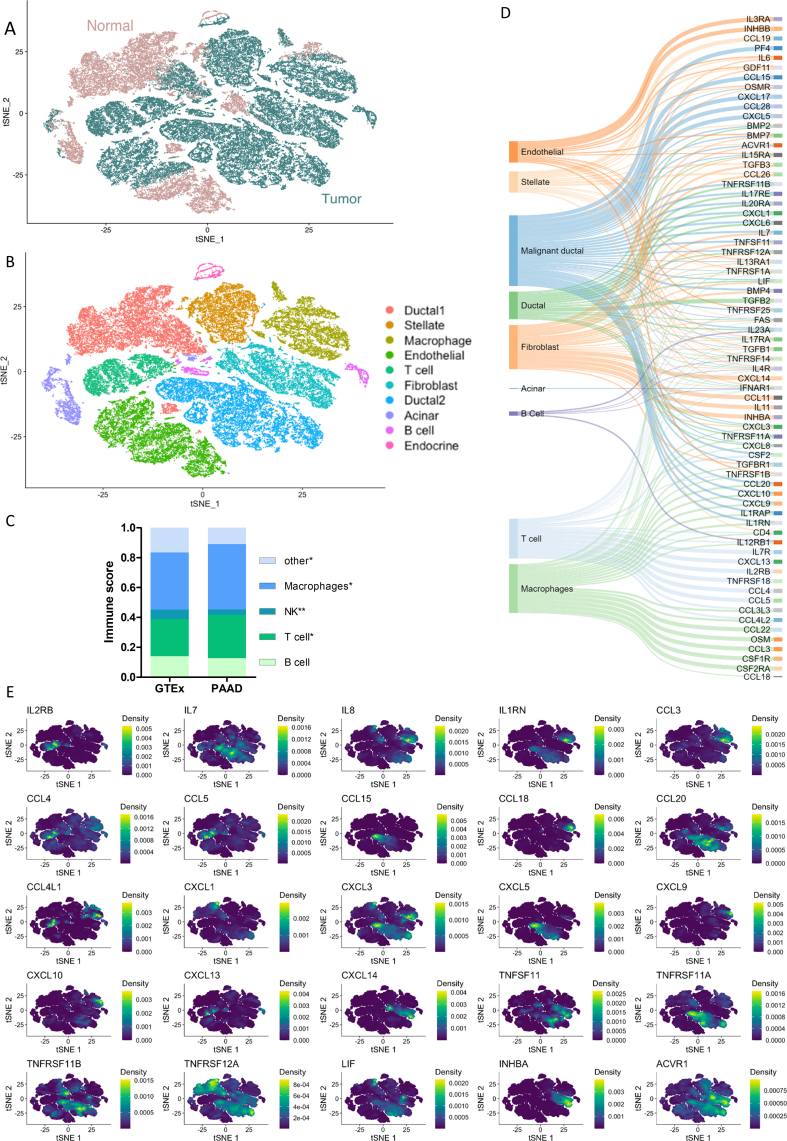
**scRNA-seq analysis identifies specific expression profiles of core-CCTF genes among different cell types in PDAC. A)** t-SNE plot of 57,530 pancreatic cells using single-cell RNA sequencing (scRNA-Seq) data from PDAC and normal-like pancreatic tissues (n=27 and n=14, respectively; CRA001160). **B)** t-SNE plot of single cells distributed in 10 cell types: acinar, ductal 1 (normal ductal cell), ductal 2 (malignant ductal cell), endothelial, fibroblast, stellate, endocrine, macrophage, B cell, and T cell. **C)** Bar graph representing the cellular proportion of Natural Killer, Macrophages, B, and T cells in PDAC from TCGA and normal pancreatic tissues from GTEx, as calculated by CIBERSORTx. ∗ p-value < 0.05, and ∗∗ p-value < 0.01. **D)** Alluvial plot demonstrating the expression of core-CCTF genes per cell type. **E)** Expression density of 26 genes belonging to the core-CCTF demonstrated in Nebulosa (v.1.7) tSNE plots in all cell types identified in single-cell analysis.

**Figure S4:**
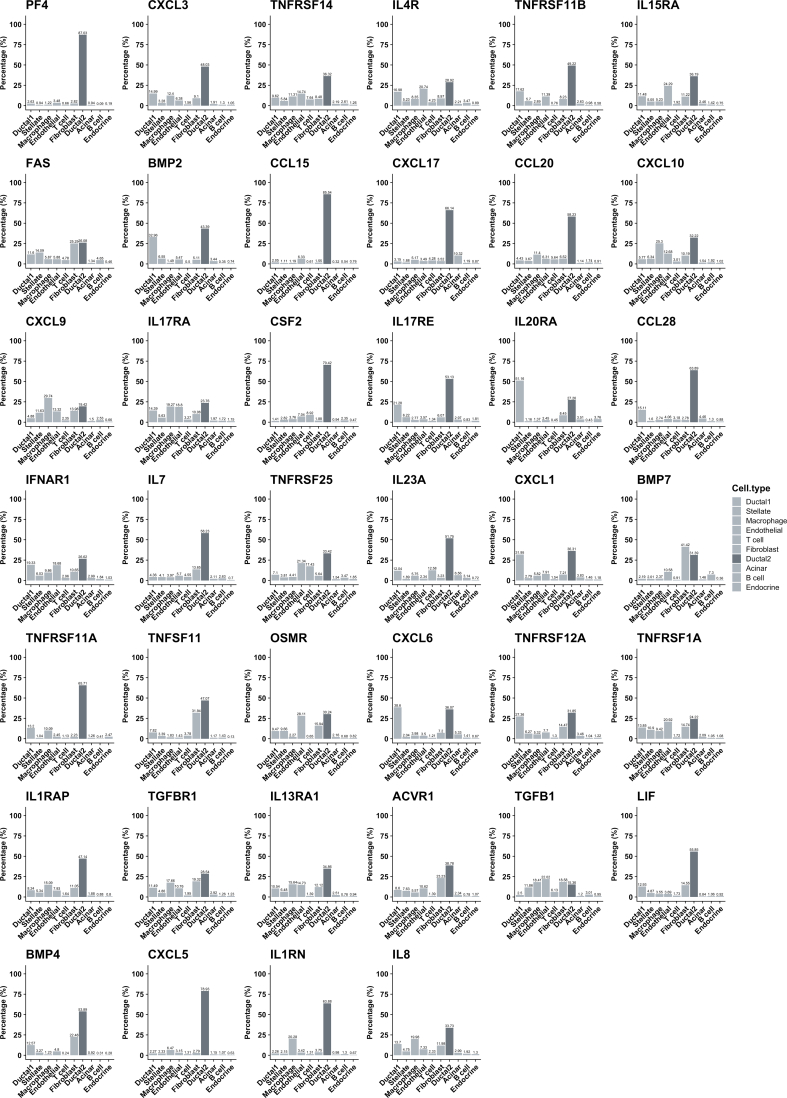
**Malignant ductal cells (Ductal2) are the primary source of core-CCTF expression.** Bar graph demonstrating the percentage of cells expressing core-CCTF in each cell type of single-cell RNA sequencing (scRNA-Seq) data from PDAC and normal-like pancreatic tissues (n=27 and n=14, respectively; CRA001160). This image was created using R (v.4.0).

**Figure S5:**
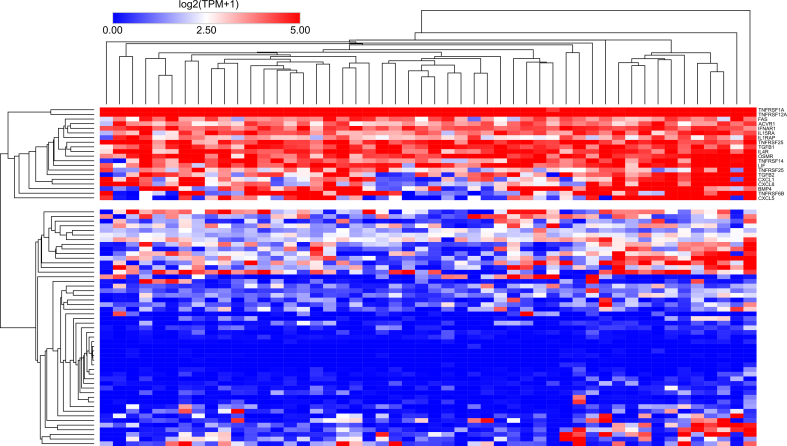
**Cancer Cell Line Encyclopedia data shows the core-CCTF expression landscape in human pancreatic cell lines.** Heatmap of core-CCTF gene z-scored expression values [log2(TPM+1)] from 50 pancreatic cancer cell lines available in the Cancer Cell Line Encyclopedia (CCLE) database. The data were obtained from the Cancer Dependency Map (DepMap; https://depmap.org/). Columns represent the cell lines and rows represent expression of the 71 core-CCTF genes. The genes highly expressed by the cell lines are depicted in the heatmap. Rows and columns were clustered using Euclidean distance. K-means analysis applied in the rows yielded two gene clusters.

**Figure S6:**
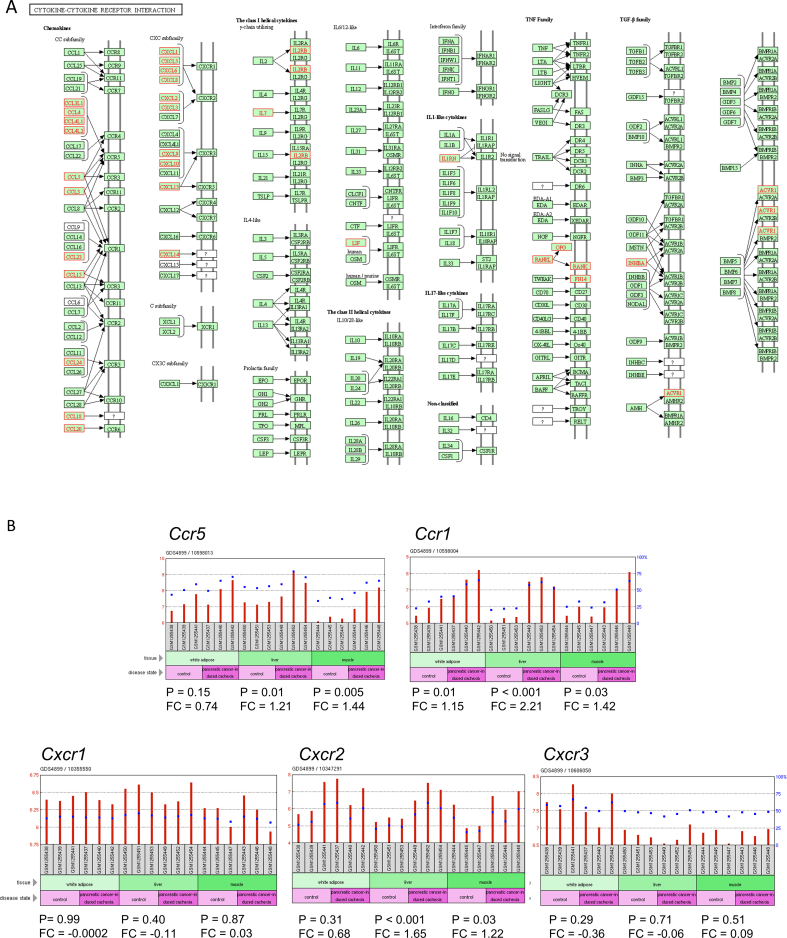
**Core-CCTF receptors CCR1 and CCR5 are increased in mice cachectic tissues. A)** Cytokine-cytokine receptor interaction (hsa04060) performed by KEGG mapper (https://www.genome.jp/kegg/mapper/). The red box indicates the genes belonging to the core-CCTF. **B)** Geoprofiles (https://www.ncbi.nlm.nih.gov/geoprofiles/) expression bar graphs demonstrating the expression of Ccr1, Ccr2, Cxcr1, Cxcr2, and Cxcr3 white adipose tissue, liver, and skeletal muscle from pancreatic cancer-induced cachexia and controls mice. Log2 Fold change (FC) and p-values were calculated using publicly expression data (GSE51931) in the GEO2R tool.
