# Supplementary material for: Data-driven identification of core tumor-secreted factors associated with cachexia prevalence
Source: Genes Dis. 2025 Oct 17;13(3):101883. doi: 10.1016/j.gendis.2025.101883 (PMC12828398; doi:10.1016/j.gendis.2025.101883)
Supplement: Multimedia component 1 [file mmc1.docx]

**Detailed description of** **Material and Methods**

**Transcriptome-based secretome analysis**

First, we downloaded of cytokine-cytokine receptor interaction (CCRI) gene list from The Kyoto Encyclopedia of Genes and Genomes (KEGG). Next, we filtered only the secreted protein-coding genes based on the human secretome list available at The Human Protein Atlas [^1^](https://www.zotero.org/google-docs/?VUIsc7) (<https://www.proteinatlas.org/humanproteome/secretome>). This list of secreted protein-coding genes was predicted by a whole-proteome scan using at least two of the three following signal peptide prediction methods: SignalP4.0, Phobius, and SPOCTOPUS. The original CCRI list had 294 genes, and the final list comprised of 263 secreted protein-coding genes, which were further analyzed.

**Pan-cancer analysis of cytokine-cytokine receptor interaction genes**

We analyzed the transcriptional profiles of these 263 genes from the CCRI pathway in 12 human cancers from The Cancer Genome Atlas, TCGA (https://portal.gdc.cancer.gov/), as previously described[^2^](https://www.zotero.org/google-docs/?vxwrRq). We compared them with matched normal tissues from TCGA and The Genotype-Tissue Expression (GTEx) Project (http://www. gtexportal.org/). We analyzed the following cancer types ranging from high to low cachexia prevalence [^3–5^](https://www.zotero.org/google-docs/?nipH5i): invasive breast carcinoma (BRCA); colon adenocarcinoma (COAD); oesophageal carcinoma (ESCA); head and neck squamous cell carcinoma (HNSC); acute myeloid leukemia (LAML); liver hepatocellular carcinoma (LIHC); lung adenocarcinoma (LUAD); lung squamous cell carcinoma (LUSC); pancreatic adenocarcinoma (PAAD); prostate adenocarcinoma (PRAD); rectum adenocarcinoma (READ); stomach adenocarcinoma (STAD). Differentially expressed genes (DEGs) between tumors and normal samples were determined by four-way ANOVA using the web-based tool Gene Expression Profiling Analysis [^6^](https://www.zotero.org/google-docs/?NgIahw) (http://gepia.cancerpku.cn/). ANOVA measures the strength of the relationship between the log‐transformed expression of a gene and all variables mentioned above between tumour and normal tissues [^7^](https://www.zotero.org/google-docs/?Dtely6). DEGs were selected using the statistical cutoffs of log2 fold change (FC) > 1 and q value < 0.01.

**Definition of a “core” of cancer cachexia tumor factors (CCTF)**

The core-CCTF represents the cytokines genes in tumor types commonly associated with cachexia. We identified the core-CCTF by selecting the secretome genes of the cytokine-cytokine receptor interaction pathway from each cancer type shared with PDAC. Next, we constructed a protein-protein interaction network of core-CCTF genes upregulated in at least five cancer types. We used STRING tool v.11, considering the following settings: experiments, databases, co-expression, neighborhood, and co-occurrence as active interaction sources. Only the highest confidence interactions were included (interaction score of at least 0.9), and the disconnected nodes were omitted from the network. PPI-enrichment p-value < 0.01 was considered significant. Visualization and data annotation of PPI networks were constructed using Cytoscape (v3.7.2) [^8^](https://www.zotero.org/google-docs/?MugK2x).

**Genomic alterations in core-CCTF and clinical outcomes in PDAC**

We analyzed the core-CCTF genomic profile in 988 PDAC patients available on cBioPortal for Cancer Genomics analysis (<https://www.cbioportal.org/>; v.5.1.7)[^9^](https://www.zotero.org/google-docs/?AGqjrk) using five different databases: International Cancer Genome Consortium (ICGC), Queensland Centre for Medical Genomics (QCMG), The Cancer Genome Atlas (TCGA), University of Texas Southwestern (UTSW), and [Clinical Proteomic Tumor Analysis Consortium](https://gdc.cancer.gov/about-gdc/contributed-genomic-data-cancer-research/clinical-proteomic-tumor-analysis-consortium-cptac) (CPTAC). We also evaluated the association of core-CCTF gene alterations with tumor hypoxia scores, overall survival (N=189 patients), and disease-free survival (N=69 patients).

**Pancreatic ductal adenocarcinoma immune tumor microenvironment (TME)**

Considering that the tumor microenvironment can be extremely relevant for cytokine production, we next investigated the cellular composition commonly observed in pancreatic cancer. CIBERSORTx tool [^10^](https://www.zotero.org/google-docs/?p0R7fK) was used to assess the immune cell type abundance of 22 cell types (LM22 matrix signature) from bulk PDAC available in TCGA and normal pancreatic tissues available in the GTEx portal (n=150 and n=165, respectively).

**Single-cell RNA sequencing from pancreatic adenocarcinoma analysis**

We reanalyzed the scRNA-seq data from Peng et al., 2019 [^11^](https://www.zotero.org/google-docs/?p8Zfzb), aiming to determine the gene expression profile of core-CCTF in 57,530 pancreatic cells using scRNA-Seq data from PDAC and normal-like pancreatic tissues (n=27 and n=14, respectively) publicly available at Genome Sequence Archive (GSA; CRA001160) [^12^](https://www.zotero.org/google-docs/?0Z6mjH) under project PRJCA001063. We also used scRNA-Seq data to analyze the expression profile of genes encoding secreted proteins. The data from raw counts were processed using the Seurat package in R (v.4.0). Cell clusters, marker genes, and Core-CCTF genes were visualized using tSNE or Nebulosa (v.1.7) dimensional reduction plots.

**Core-CCTF expression analysis using Cancer Cell Line Encyclopedia data**

To confirm the gene expression of Core-CCFT genes in malignant cells, we evaluated the gene expression data (log2 transformed transcripts per million - TPM, using pseudo-count of 1) of the 71 core-CCTF in 50 pancreatic cancer cell lines from the Cancer Cell Line Encyclopedia (CCLE) portal (<https://portals.broadinstitute.org/ccle>) [^13^](https://www.zotero.org/google-docs/?WIkFu3) were downloaded using the Cancer Dependency Map (DepMap; https://depmap.org/) data portal (Expression 21Q2 Public datasets) [^14^](https://www.zotero.org/google-docs/?6VDGQe).

**Tumor and cachexia targeted tissue communication mediated by core-CCTF**

To check how the core-CCTF from PDAC tumor cells can induce alterations in muscle, adipose, liver, brain, and bone human primary cells, we next predicted ligands existing in the core-CCTF list and the gene expression of their receptors in cells, tissues, or organs affected in cancer cachexia. We used the ligand-receptor consensus list by Ramilowski et al., 2015 [^15^](https://www.zotero.org/google-docs/?LWdIVL) to verify the expression of the CCTF receptors in human primary cells from the FANTOM5 project [^16^](https://www.zotero.org/google-docs/?xfPTVP). The KEGG mapper tool (https://www.genome.jp/kegg/mapper/search.html) was used to visualize the core-CCTF in the CCRI pathway and their corresponding receptors. We selected the receptors with more than three ligands for further investigation. Furthermore, the microarray data from GSE51931 was used to measure the expression of these selected receptor tissues (skeletal, adipose, and liver) in a pancreatic cancer cachexia model [^17^](https://www.zotero.org/google-docs/?GWeiq4). The gene expression analysis compared cachectic tissues and controls using the GEO2R tool (<http://www.ncbi.nlm.nih.gov/geo/geo2r/>).

**Data representation**

Heatmaps were created using the web tool Morpheus (<https://software.broadinstitute.org/morpheus>). An alluvial diagram connecting the ligands to their respective receptor was generated using the SankeyMATIC online tool (<http://sankeymatic.com/>).

Supporting Literature for the Discussion

Discussion on the importance of IL-8 for cancer cachexia: IL-8 is a tumor-derived factor directly associated with muscle wasting in lung and pancreatic cancers 18, 19, and its neutralization with blocking antibodies prevented in vitro muscle atrophy 19.

Discussion on the tumor microenvironment: Liu et al. have shown that macrophages potentiate pancreatic cancer–induced muscle wasting by promoting TWEAK secretion from tumor cells, and that depletion of macrophages can reverse tumor-driven muscle degradation. Macrophages activate malignant cells through the CCL5/TRAF6/NF-κB pathway, leading to non-autonomous secretion of TWEAK 20.

**References**

[1. Thul PJ, Åkesson L, Wiking M, et al. A subcellular map of the human proteome. *Science*. 2017;356(6340):eaal3321. doi:10.1126/science.aal3321](https://www.zotero.org/google-docs/?ib7uHC)

[2. Freire PP, Fernandez GJ, Moraes D, et al. The expression landscape of cachexia‐inducing factors in human cancers. *J Cachexia Sarcopenia Muscle*. 2020;11(4):947-961. doi:10.1002/jcsm.12565](https://www.zotero.org/google-docs/?ib7uHC)

[3. Baracos VE, Martin L, Korc M, Guttridge DC, Fearon KCH. Cancer-associated cachexia. *Nat Rev Dis Primer*. 2018;4(1):17105. doi:10.1038/nrdp.2017.105](https://www.zotero.org/google-docs/?ib7uHC)

[4. Hébuterne X, Lemarié E, Michallet M, de Montreuil CB, Schneider SM, Goldwasser F. Prevalence of Malnutrition and Current Use of Nutrition Support in Patients With Cancer. *J Parenter Enter Nutr*. 2014;38(2):196-204. doi:10.1177/0148607113502674](https://www.zotero.org/google-docs/?ib7uHC)

[5. Pressoir M, Desné S, Berchery D, et al. Prevalence, risk factors and clinical implications of malnutrition in French Comprehensive Cancer Centres. *Br J Cancer*. 2010;102(6):966-971. doi:10.1038/sj.bjc.6605578](https://www.zotero.org/google-docs/?ib7uHC)

[6. Tang Z, Li C, Kang B, Gao G, Li C, Zhang Z. GEPIA: a web server for cancer and normal gene expression profiling and interactive analyses. *Nucleic Acids Res*. 2017;45(W1):W98-W102. doi:10.1093/nar/gkx247](https://www.zotero.org/google-docs/?ib7uHC)

[7. Malod-Dognin N, Petschnigg J, Windels SFL, et al. Towards a data-integrated cell. *Nat Commun*. 2019;10(1):805. doi:10.1038/s41467-019-08797-8](https://www.zotero.org/google-docs/?ib7uHC)

[8. Shannon P. Cytoscape: A Software Environment for Integrated Models of Biomolecular Interaction Networks. *Genome Res*. 2003;13(11):2498-2504. doi:10.1101/gr.1239303](https://www.zotero.org/google-docs/?ib7uHC)

[9. Gao J, Aksoy BA, Dogrusoz U, et al. Integrative Analysis of Complex Cancer Genomics and Clinical Profiles Using the cBioPortal. *Sci Signal*. 2013;6(269). doi:10.1126/scisignal.2004088](https://www.zotero.org/google-docs/?ib7uHC)

[10. Chen B, Khodadoust MS, Liu CL, Newman AM, Alizadeh AA. Profiling Tumor Infiltrating Immune Cells with CIBERSORT. In: von Stechow L, ed. *Cancer Systems Biology*. Vol 1711. Methods in Molecular Biology. Springer New York; 2018:243-259. doi:10.1007/978-1-4939-7493-1_12](https://www.zotero.org/google-docs/?ib7uHC)

[11. Peng J, Sun BF, Chen CY, et al. Single-cell RNA-seq highlights intra-tumoral heterogeneity and malignant progression in pancreatic ductal adenocarcinoma. *Cell Res*. 2019;29(9):725-738. doi:10.1038/s41422-019-0195-y](https://www.zotero.org/google-docs/?ib7uHC)

[12. Wang Y, Song F, Zhu J, et al. GSA: Genome Sequence Archive *. *Genomics Proteomics Bioinformatics*. 2017;15(1):14-18. doi:10.1016/j.gpb.2017.01.001](https://www.zotero.org/google-docs/?ib7uHC)

[13. Barretina J, Caponigro G, Stransky N, et al. The Cancer Cell Line Encyclopedia enables predictive modelling of anticancer drug sensitivity. *Nature*. 2012;483(7391):603-607. doi:10.1038/nature11003](https://www.zotero.org/google-docs/?ib7uHC)

[14. Ghandi M, Huang FW, Jané-Valbuena J, et al. Next-generation characterization of the Cancer Cell Line Encyclopedia. *Nature*. 2019;569(7757):503-508. doi:10.1038/s41586-019-1186-3](https://www.zotero.org/google-docs/?ib7uHC)

[15. Ramilowski JA, Goldberg T, Harshbarger J, et al. A draft network of ligand–receptor-mediated multicellular signalling in human. *Nat Commun*. 2015;6(1):7866. doi:10.1038/ncomms8866](https://www.zotero.org/google-docs/?ib7uHC)

[16. Lizio M, Harshbarger J, Shimoji H, et al. Gateways to the FANTOM5 promoter level mammalian expression atlas. *Genome Biol*. 2015;16(1):22. doi:10.1186/s13059-014-0560-6](https://www.zotero.org/google-docs/?ib7uHC)

[17. Gilabert M, Calvo E, Airoldi A, et al. Pancreatic Cancer-Induced Cachexia Is Jak2-Dependent in Mice: Jak2-DEPENDENT CACHEXIA. *J Cell Physiol*. 2014;229(10):1437-1443. doi:10.1002/jcp.24580](https://www.zotero.org/google-docs/?ib7uHC)

18. Cury SS, de Moraes D, Freire PP, et al. Tumor Transcriptome Reveals High Expression of IL-8 in Non-Small Cell Lung Cancer Patients with Low Pectoralis Muscle Area and Reduced Survival. *Cancers* (Basel). 2019;11(9). doi:10.3390/cancers11091251

19. Callaway CS, Delitto AE, D’Lugos AC, et al. IL-8 Released from Human Pancreatic Cancer and Tumor-Associated Stromal Cells Signals through a CXCR2-ERK1/2 Axis to Induce Muscle Atrophy. *Cancers*. 2019;11(12):1863. doi:10.3390/cancers11121863

20. Liu M, Ren Y, Zhou Z, et al. The crosstalk between macrophages and cancer cells potentiates pancreatic cancer cachexia. *Cancer Cell*. 2024;42(5):885-903.e4. doi:10.1016/j.ccell.2024.03.009

**Supplementary figure captions**

**Figure S1** Pearson correlation-based clustering analysis of core-CCTFs and expression profiles across tumor types. The heatmap on the left shows co-expression modules identified among the 71 core-CCTFs, with the highlighted clusters representing groups of genes with the highest correlations. On the right, the bar plots represent the average log_2_ expression values of each cluster across the 12 analyzed tumor types, revealing distinct transcriptional patterns associated with cachexia prevalence.

**Figure S2** Genomic alterations on core-CCTF genes are associated with high hypoxia scores and lower disease-free survival. **(A)** Oncoprint demonstrating genomic alterations of 71 core-CCTF genes in 988 pancreatic adenocarcinoma patients. **(B)** Box plot representing hypoxia scores calculated by the cBioPortal tool using three metrics (Buffa, Ragnum, and Winter). The statistical significance was analyzed using the Wilcoxon test. *P*-values were corrected using the Benjamini-Hochberg FDR procedure. **(C)** Kaplan-Meier disease-free survival curve for 69 PDAC patients. The resulting *P*-values for the log-rank test are shown. **(D)** Kaplan-Meier overall survival curve for 189 PDAC patients. The resulting *P*-values for the log-rank test are shown. Altered group: PDAC patients with gene alterations in core-CCTF genes. Unaltered group: PDAC patients with wild-type core-CCTF genes. Figure generated using the cBioPortal web tool (https://www.cbioportal.org/) using patients' available information from five different databases: International Cancer Genome Consortium (ICGC), Queensland Centre for Medical Genomics (QCMG), The Cancer Genome Atlas (TCGA), University of Texas Southwestern (UTSW), and [Clinical Proteomic Tumor Analysis Consortium](https://gdc.cancer.gov/about-gdc/contributed-genomic-data-cancer-research/clinical-proteomic-tumor-analysis-consortium-cptac) (CPTAC).

**Figure S3** Single-cell RNA sequencing (scRNA-seq) analysis identifies specific expression profiles of core-CCTF genes among different cell types in PDAC. **(A)** t-SNE plot of 57,530 pancreatic cells using scRNA-seq data from PDAC and normal-like pancreatic tissues (*n* = 27 and *n* = 14, respectively; CRA001160). **(B)** t-SNE plot of single cells distributed in 10 cell types: acinar cell, ductal 1 (normal ductal cell), ductal 2 (malignant ductal cell), endothelial cell, fibroblast, stellate cell, endocrine cell, macrophage, B cell, and T cell. **(C)** Bar graph representing the cellular proportion of natural killer cells, macrophages, B cells, and T cells in PDAC from TCGA and normal pancreatic tissues from GTEx, as calculated by CIBERSORTx. ^*^*P* < 0.05 and ^**^*P* < 0.01 **(D)** Alluvial plot demonstrating the expression of core-CCTF genes per cell type. **(E)** Expression density of 26 genes belonging to the core-CCTF was demonstrated in Nebulosa (v.1.7) t-SNE plots in all cell types identified in single-cell analysis.

**Figure S4** Malignant ductal cells (Ductal2) are the primary source of core-CCTF expression. Bar graph demonstrating the percentage of cells expressing core-CCTF in each cell type of single-cell RNA sequencing data from PDAC and normal-like pancreatic tissues (*n* = 27 and *n* = 14, respectively; CRA001160). This image was created using R (v.4.0).

**Figure S5** Cancer Cell Line Encyclopedia data shows the core-CCTF expression landscape in human pancreatic cell lines. Heatmap of core-CCTF gene z-scored expression values [log_2_(TPM+1)] from 50 pancreatic cancer cell lines available in the Cancer Cell Line Encyclopedia (CCLE) database. The data were obtained from the Cancer Dependency Map (DepMap; https://depmap.org/). Columns represent the cell lines, and rows represent the expression of the 71 core-CCTF genes. The genes highly expressed by the cell lines are depicted in the heatmap. Rows and columns were clustered using Euclidean distance. K-means analysis applied to the rows yielded two gene clusters.

**Figure S6** Core-CCTF receptors CCR1 and CCR5 are increased in mice cachectic tissues. **(A)** Cytokine-cytokine receptor interaction ([hsa04060](https://www.genome.jp/dbget-bin/www_bget?hsa04060)) performed by KEGG mapper (<https://www.genome.jp/kegg/mapper/>). The red box indicates the genes belonging to the core-CCTF. **(B)** Geoprofiles (https://www.ncbi.nlm.nih.gov/geoprofiles/) expression bar graphs demonstrating the expression of *Ccr1*, *Ccr2*, *Cxcr1*, *Cxcr2*, and *Cxcr3* in white adipose tissue, liver, and skeletal muscle from pancreatic cancer-induced cachexia and control mice. Log_2_fold-change (FC) and *P*-values were calculated using publicly available expression data (GSE51931) in the GEO2R tool.
